# Practical Recommendations for Diagnosis and Management of Respiratory Muscle Weakness in Late-Onset Pompe Disease

**DOI:** 10.3390/ijms17101735

**Published:** 2016-10-17

**Authors:** Matthias Boentert, Hélène Prigent, Katalin Várdi, Harrison N. Jones, Uwe Mellies, Anita K. Simonds, Stephan Wenninger, Emilia Barrot Cortés, Marco Confalonieri

**Affiliations:** 1 Department of Sleep Medicine and Neuromuscular Disorders, Münster University Hospital, Münster 48149, Germany; 2 Physiology Department and Neuromuscular Home Ventilation Unit, Raymond Poincaré University Hospital, Garches 92380, France; helene.prigent@aphp.fr; 3 Respiratory Rehabilitation and Sleep Center, Törökbálint Chest Hospital, Törökbálint 2045, Hungary; katalinvardi@gmail.com; 4 Department of Surgery, Duke University, Division of Speech Pathology & Audiology, Durham, NC 27710, USA; harrison.jones@duke.edu; 5 Department of Pediatric Pulmonology and Sleep Medicine, University of Duisburg-Essen, Children’s Hospital, Essen 45147, Germany; uwe.mellies@uk-essen.de; 6 Academic and Clinical Department of Sleep and Breathing, Royal Brompton & Harefield NHS Foundation Trust, London SW3 6NP, UK; a.simonds@rbht.nhs.uk; 7 Friedrich-Baur-Institute, Department of Neurology, Ludwig-Maximilians-University, Munich 80336, Germany; stephan.wenninger@med.uni-muenchen.de; 8 Medical-Surgical Unit of Respiratory Diseases, University Hospital Virgen del Rocio, Seville 41013, Spain; emilia.barrot.sspa@juntadeandalucia.es; 9 Department of Pulmonology, University Hospital of Cattinara, Trieste 34149, Italy; marco.confalonieri@aots.sanita.fvg.it

**Keywords:** neuromuscular disorders, Pompe disease, respiratory muscle weakness, mechanical ventilation, cough assistance

## Abstract

Pompe disease is an autosomal-recessive lysosomal storage disorder characterized by progressive myopathy with proximal muscle weakness, respiratory muscle dysfunction, and cardiomyopathy (in infants only). In patients with juvenile or adult disease onset, respiratory muscle weakness may decline more rapidly than overall neurological disability. Sleep-disordered breathing, daytime hypercapnia, and the need for nocturnal ventilation eventually evolve in most patients. Additionally, respiratory muscle weakness leads to decreased cough and impaired airway clearance, increasing the risk of acute respiratory illness. Progressive respiratory muscle weakness is a major cause of morbidity and mortality in late-onset Pompe disease even if enzyme replacement therapy has been established. Practical knowledge of how to detect, monitor and manage respiratory muscle involvement is crucial for optimal patient care. A multidisciplinary approach combining the expertise of neurologists, pulmonologists, and intensive care specialists is needed. Based on the authors’ own experience in over 200 patients, this article conveys expert recommendations for the diagnosis and management of respiratory muscle weakness and its sequelae in late-onset Pompe disease.

## 1. Introduction

Empirical data on the management of respiratory muscle weakness in adults with Pompe disease are scarce. Clinical recommendations for the care of patients with Pompe disease do not specifically focus on respiratory issues and standard practice is largely based on guidelines covering chronic respiratory failure in neuromuscular disease (NMD) in general [1,2,3,4]. A recent, more disease-specific publication provides only limited coverage of practical aspects such as cough assistance, respiratory muscle training, and ventilation techniques [5]. Lack of treatment guidelines has led to the adoption of heterogeneous, often local standards of practice. The low prevalence of Pompe disease makes it difficult to establish a national or international consensus. In order to create comprehensive recommendations for diagnosis and management of respiratory muscle weakness, the Pompe Disease Respiratory Care Working Group was formed in 2013, bringing together experts from various clinical disciplines. The following recommendations provide practical, technical, and, wherever possible, disease-specific guidance for physicians who care for patients with late-onset Pompe disease (LOPD).

## 2. Background

Pompe disease is an autosomal-recessive lysosomal storage disorder caused by α-1,4-glucosidase (GAA) enzyme deficiency. Prevalence ranges between 1:40,000 and 1:100,000 [6]. GAA dysfunction results in accumulation of glycogen in skeletal and smooth muscle cells, hepatocytes, endothelial cells, and central nervous system neurons [7]. GAA activity below 1% is associated with early-infantile disease onset, cardiomyopathy, cardiorespiratory failure, and early death if enzyme replacement therapy (ERT) is not initiated [8]. Partial reduction of GAA enzyme activity is associated with late-childhood, juvenile, or adult disease onset, which is mainly characterized by progressive weakness of the limb girdle and axial muscles. In this article, the term “late-onset Pompe disease” (LOPD) comprises virtually all disease subtypes other than early-infantile. In LOPD, respiratory muscle dysfunction may precede limb girdle weakness, and ventilatory support is indicated prior to wheelchair dependence in about one-third of patients [9]. Affected respiratory muscles comprise the diaphragm in particular, but also the upper airway, and intercostal and abdominal muscles in advanced disease [10,11,12]. Recent evidence shows that diaphragmatic dysfunction cannot only be attributed to myopathic changes but also to accumulation of glycogen in cervical anterior horn cells and alterations of both phrenic nerve fibers and neuromuscular junctions, respectively [13,14]. Thus, therapeutic effects of ERT on diaphragmatic function may be limited since partial clearance of glycogen from myocytes may be counterbalanced by persisting neuronal pathology. However, long-term ERT has been shown to slightly improve forced vital capacity in patients with LOPD [15,16]. In untreated patients with LOPD chronic respiratory failure slowly develops in more than 70% with a mean annual decline of the forced vital capacity (FVC) of about 1.5% [17]. However, disease progression and diaphragmatic involvement both are highly variable in LOPD patients with and without ERT making it obligatory to assess and monitor respiratory muscle strength on an individual basis. Since impairment of respiratory muscle function is still ongoing in one third of adults on ERT [18] anticipatory management strategies aim to improve inspiratory muscle function and to early identify the need for ventilatory support. Work-up of clinically apparent sleep disturbances is crucial since symptoms of sleep-disordered breathing (SDB) may indicate respiratory muscle weakness [19]. In addition, impairment of cough and airway clearance is a common finding in patients with respiratory muscle dysfunction since both inspiratory capacity and expiratory force are reduced.

## 3. Assessment of Respiratory Muscle Function

### 3.1. Clinical Presentation

Daytime symptoms suggestive of respiratory muscle weakness include dyspnea on exertion or rest, dyspnea on immersion in water, and reduced physical capacity. Sleep-related symptoms include orthopnea, sleep disruption, morning headache, daytime hypersomnolence, and fatigue. Alveolar hypoventilation is more likely to occur in the supine position and during rapid eye movement (REM) sleep when muscle tone is physiologically decreased. Impaired cough predisposes patients to deep aspiration, mucus obstruction, and pulmonary infections. Reccurent respiratory infections or prolonged recovery from such illnesses may reflect respiratory muscle weakness even before dyspnea or sleep-disordered symptoms become apparent. Diaphragmatic dysfunction is usually accompanied by weakness of the axial musculature and proximal limb muscles leading to hyperlordotic posture and Trendelenburg’s gait.

### 3.2. Screening Questionnaires

Disease-specific screening questionnaires for symptoms of respiratory muscle weakness do not exist. Validated screening questionnaires including the Medical Research Council (MRC) Breathlessness Scale [20] the Baseline and Transition Dyspnea Index [21] or the Modified Borg Scale [22] can be used to evaluate dyspnea. However, none of these instruments has been validated for neuromuscular disease or LOPD in particular. Only the Sleep-Disordered Breathing in Neuromuscular Disease Questionnaire (SiNQ-5) [23] has been specifically designed for patients with NMD but has not widely been used.

### 3.3. Clinical Examination

Respiratory rate, speech, and activation of auxiliary respiratory muscles should be assessed in the upright and supine position. In the latter, paradoxical breathing may be present reflecting advanced diaphragmatic weakness. With disease progression, patients may be unable to sustain the supine position without ventilatory support, and long-standing hypercapnia may cause bilateral ankle edema or clinical signs of cor pulmonale, although this is rare [24].

### 3.4. Measurement of Respiratory Muscle Function

#### 3.4.1. Pulmonary Function Tests (PFTs)

Normal values for PFTs have been published [25,26]. PFTs usually show restriction with a decrease in vital capacity (VC) and total lung capacity (TLC). VC is defined as the maximal volume a patient can exhale or inhale, respectively. Notably, VC does not directly reflect respiratory muscle strength but depends on inspiratory and expiratory muscle function alongside with the structural features of the chest wall and the lungs. Various methods of VC testing have been described including VC, forced and slow VC (all expiratory) or inspiratory VC (IVC). However, these maneuvers all assess the same parameter in any given individual. VC may differ from FVC only in patients with chronic obstructive lung disease, and SVC (slow vital capacity) may be superior to FVC if there is a risk of air leakage due to impaired lip closure or insufficient sealing of the nasopharynx by the velum, respectively. If respiratory muscle weakness is just emerging, an isolated decrease of VC can be observed while TLC is still normal [27]. VC should be evaluated in both the upright and supine positions, and a >20% drop indicates significant diaphragmatic weakness [28]. Decreased IVC is predictive of either SDB (<60%), or nocturnal hypoventilation (<40%) [28,29] and an IVC < 25% has been shown to be associated with diurnal respiratory failure in NMD [30]. Expiratory muscle weakness may significantly alter the upright VC and the expected positional drop of the VC.

#### 3.4.2. Peak Cough Flow (PCF)

PCF can be obtained using a hand-held device. Healthy adults show PCF > 400 L/min. A PCF < 160 L/min reflects inadequate airway clearance. Values between 160 and 270 L/min predict susceptibility to respiratory tract infections [31]. PCF should be routinely measured if PCF was <270 L/min once (or <160 L/min during acute exacerbation) and if impaired cough is clinically apparent.

#### 3.4.3. Manometry

Maximal inspiratory pressure (MIP), sniff nasal inspiratory pressure (SNIP), and maximal expiratory pressure (MEP) are volitional measures of respiratory muscle strength. Changes of MIP and MEP are often detectable before VC and TLC decline. MIP and MEP testing should be performed according to accepted standards [32]. Reference values and equations have been published [33,34]. All tests should be repeated at least three times. MIP and SNIP are considered complementary rather than interchangeable, and when both techniques are applied, the highest value measured should be recorded [35]. The technique that is performed best by a patient should be used for follow-up.

#### 3.4.4. Non-Volitional Tests

Non-volitional measures of diaphragmatic strength include transdiaphragmatic twitch pressure (Pdi) and twitch mouth pressure (Pmo) after magnetic phrenic nerve stimulation [36]. Since Pdi is highly correlated to VC, MIP, and MEP in adults with Pompe disease, invasive assessment of respiratory muscle strength is not advisable in routine clinical settings. Phrenic nerve conduction studies and needle electromyogram of the diaphragm may yield further information on diaphragmatic function but have not been evaluated in patients with LOPD. Imaging techniques that help assess diaphragmatic function include transmission radiography, ultrasound and magnetic resonance (MR) tomography. Thoracic radiography is widely available but has limited sensitivity and does not allow for quantification of RMW (respiratory muscle weakness) [32]. Ultrasound can reveal both impaired excursions and reduced thickness of the diaphragm [37]. MR imaging may potentially be useful to detect altered diaphragm kinetics in neuromuscular disease including LOPD [38], however, normative values and clinical validation are still lacking, thus preventing its use in clinical routine to date.

In summary, various complementary methods of respiratory muscle assessment are available and should be utilized to facilitate the diagnosis of either SDB or daytime respiratory failure in ventilator-free patients. FVC, MIP, and MEP have been shown predict the need for mechanical ventilation in LOPD [39] and should be preferred for primary assessment. In addition, the above measures allow for monitoring of disease progression and proper timing of follow-up visits in both ventilated and non-ventilated patients (Table 1). For practical reasons, measurement of FVC or IVC in the upright and supine position is most likely to be readily available, and further methods may be provided only by specialized centers.

Testing should be carried out by trained staff at baseline (diagnosis) and at least once a year for routine follow-up. Test intervals should be shorter (e.g., every 3–6 months) if signs and symptoms of RMW are present or if acute exacerbation has just occurred. A nasal clip is obligatory except for SNIP testing. Severe RMW is indicated by significant reduction of VC, ERV, or MIP/MEP/SNIP. TLC, RV, and IC are not helpful for differentiation of severe and mild/moderate RMW.

### 3.5. Sleep Studies

**Figure 1:**
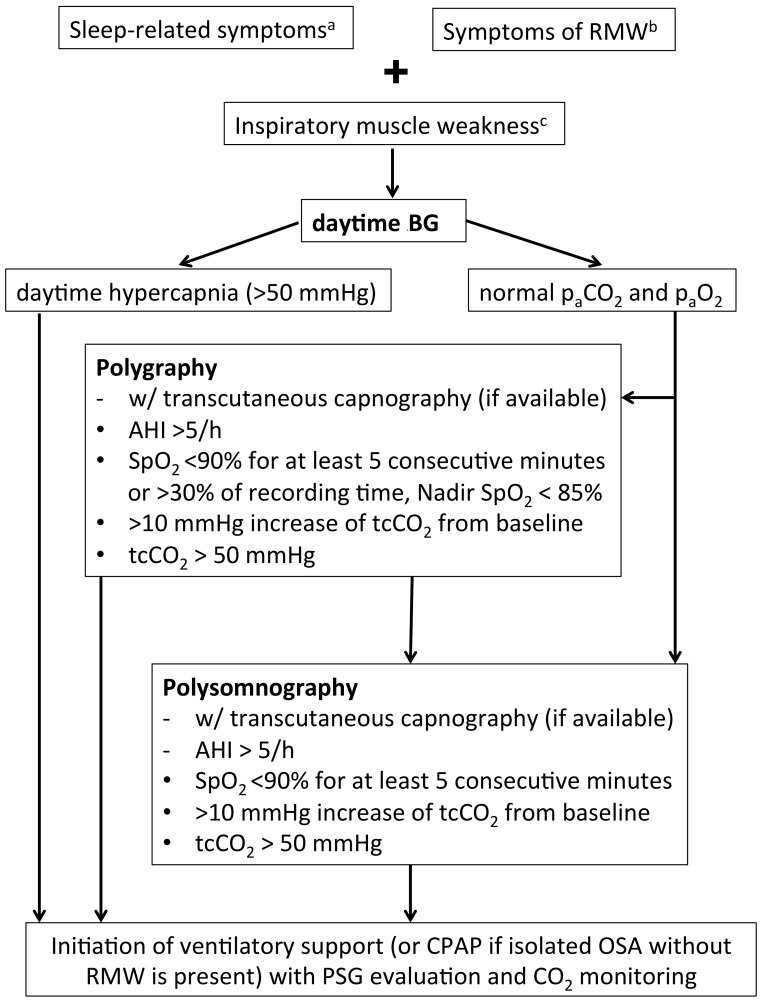
Recommendations for sleep studies in patients with LOPD. Isolated nocturnal tachypnea or lone increase of base excess on early-morning blood gas analysis may both be indicative of nocturnal hypoventilation but do not justify ventilatory support. However, both scenarios should give rise to monitor patients in shorter intervals. ^a^ sleep disruption, morning headache, daytime hypersomnolence; ^b^ dyspnea, orthopnea; ^c^ VC < 50% predicted, VC postural drop > 40%, MIP < 60 cm H_2_O, SNIP < 40 cm H_2_O. VC, vital capacity; RMW, respiratory muscle weakness; paCO_2_, carbon dioxide tension; paO_2_, oxygen tension; AHI, apnea hypopnea index; SpO_2_, oxygen saturation; tcCO_2_, transcutaneous carbon dioxide tension; CPAP, continuous positive airway pressure; OSA, obstructive sleep apnea; PSG, polysomnography; SNIP, sniff nasal inspiratory pressure; MIP maximum inspiratory pressure.

Sleep-related symptoms should be assessed using standard questionnaires such as the Epworth Sleepiness Scale [40], the Pittsburgh Sleep Quality Index [41], and the Fatigue Severity Scale [42] since sleep disruption by nocturnal hypercapnia may contribute to physical exhaustion. However, self-reported sleep outcomes do not specifically indicate SDB. Nocturnal hypercapnia can be detected by nighttime blood gases or by the presence of an increased base excess (BE) during the day reflecting compensatory retention of bicarbonate. A base excess >4 mmol/L has been shown to be a strong predictor of nocturnal hypoventilation in patients with Duchenne muscular dystrophy [43]. If daytime pCO_2_ and BE are normal, early morning blood gases may be more sensitive to unmask nocturnal hypercapnia by showing either elevated or high-normal pCO_2_, or an increased BE. Normal daytime blood gases do not exclude respiratory muscle weakness because compensatory tachypnea may normalize or even decrease daytime pCO_2_. Pulse oximetry (PO) detects nocturnal oxygen desaturation. Hypoventilation is indicated by peripheral oxygen saturation (SpO_2_) < 90% for five consecutive minutes or more, a minimal SpO_2_ < 85%, or SpO_2_ < 90% for at least 30% of recording time. However, PO may show normal results if hypoventilation is either mild or short-lasting. In these cases, hypoventilation may be only unmasked by carbon dioxide (CO_2_) measurement. For this reason, PO alone is not recommended as a screening tool for SDB in patients with NMD [44]. Combination of PO and blood gas analysis may be considered clinically sufficient, practical and cost-effective if more sophisticated sleep studies are not available or if patients cannot be seen in a specialized center. Transcutaneous capnography allows non-invasive real-time monitoring of peripheral carbon dioxide tension (tcCO_2_). It directly reflects alveolar ventilation and detects periods of nocturnal hypoventilation (tcCO_2_ > 50 mmHg) with high sensitivity. Constant increase of tcCO_2_ during the course of the night reflects decreased respiratory muscle endurance. Cardiorespiratory polygraphy (PG) comprises registration of oxygen saturation, nasal and oral airflow, respiratory effort, heart rate, and body position. It allows for identification of hypopneas, central and obstructive apneas, nocturnal tachypnea, and prolonged episodes of hypoventilation reflected by persistent desaturation in the absence of upper airway obstruction. PG should be preferred as a screening tool if concomitant obstructive sleep apnea is suspected. Combination with blood gas analysis or capnography is strongly recommended. Cardiorespiratory polysomnography (PSG) combines polygraphy, electrooculogram, and electroencephalogram with optional videography. It allows for correlation of any respiratory event with sleep and sleep stages. Combination of PSG and CO_2_ monitoring detects REM sleep-associated hypercapnia as the earliest sign of nocturnal hypoventilation, thus yielding the highest sensitivity in detecting SDB. Reduced REM sleep is a frequent finding in patients with significant respiratory muscle weakness [29]. Full PSG is generally recommended for baseline evaluation of sleep (Figure 1) [44].

### 3.6. Daytime Blood Gas Analysis

Daytime blood gases do not necessarily have to be drawn from arterial blood since this procedure may often be restricted to specialized respiratory units or laboratories, respectively. Blood gases taken from the arterialized earlobe or serum bicarbonate from a routine electrolyte panel are sufficient and more readily available. Serum bicarbonate does reflect chronic alveolar hypoventilation in NMD and other hypoventilation syndromes [45].

## 4. Management

### 4.1. Mechanical Ventilation

Mechanical ventilation has improved survival in NMD with progressive respiratory involvement [46]. It comprises non-invasive ventilation (NIV) and tracheostomy invasive ventilation (TIV). NIV has been shown to effectively correct alveolar hypoventilation and alleviate sleep-related symptoms in patients with both infantile-onset and juvenile/adult onset Pompe disease [47,48,49]. Mechanical ventilation corrects nocturnal hypercapnia and re-sensitizes respiratory centers to CO_2_ by persistently decreasing plasma bicarbonate levels [50]. Intermittent mechanical ventilation may influence strength and endurance of respiratory muscles. In addition, it promotes rib cage and lung expansion which may help prevent atelectasis, ventilation-perfusion mismatch, and infections. Ventilator-induced diaphragmatic dysfunction due to long-term ventilation has been described in critically ill patients [51] but has not been investigated in patients with NMD receiving intermittent ventilatory support.

#### 4.1.1. Non-Invasive Ventilation (NIV)

Indication criteria for NIV in chronic respiratory failure due to NMD can be adopted from existing guidelines (Table 2) [32]. Conditions promoting or worsening respiratory failure should be ruled out or treated adequately. Ventilation mode, ventilator settings, and interfaces should be personalized by experienced personnel according to sleep study results and individual needs. In a few cases sleep studies may be expendable if daytime hypercapnia or very severe respiratory muscle weakness is present, but the presence of concomitant obstructive sleep apnea should not be missed regarding its relevance for pressure settings. Humidification should always be offered. Nasal masks may be more comfortable, but oronasal interfaces reduce air leakage, and even mouthpieces have successfully been used in patients with NMD on long-term NIV. Titration of ventilator settings and treatment evaluation should be performed using PSG and CO_2_ monitoring. For routine follow-up, serum bicarbonate on both NIV and spontaneous breathing may be sufficient if NIV is used regularly and patient comfort is good [52]. Blood gas analysis and sleep studies including capnometry are necessary if serum bicarbonate is elevated or in case of patient discomfort, recurring symptoms of SDB, or marked progression of respiratory muscle weakness.

#### 4.1.2. Tracheostomy Invasive Ventilation (TIV)

Indications and contraindications for invasive ventilation are depicted in Table 2. In case of NIV failure one should address inadequate ventilator settings, mucus obstruction, or mask intolerance before tracheostomy is considered. For patients with advanced disease and tetraplegia or with serious comorbidities, it may be appropriate to offer palliative care instead of TIV, especially if ceiling of care is part of an advanced directive. Long-term TIV should be administered by an experienced center with trained home support technicians. Surgical tracheostomy should be preferred. Continous mandatory ventilation is obligatory including humidification, and uncuffed or deflated tracheostomy tubes should be used to support speech and swallowing. Patients should be equipped with two ventilators and adequate devices necessary for oxymetry, assisted cough, and suction. Caregivers should receive both comprehensive training and 24/7 online technical support. Follow-up of patients receiving TIV includes blood gas analysis under ventilation and spontaneous breathing (if possible), oxymetry, and nocturnal CO_2_ monitoring, if appropriate. In-hospital evaluation including PFT, blood gas analysis, and sleep studies is recommended in case of patient discomfort, symptoms suggestive of persistent hypercapnia, or acute respiratory failure. For patients on long-term NIV or TIV, a second ventilator should be provided if mechanical ventilation exceeds 16 h a day, and a battery-powered device should be prescribed in order to maintain mobility (e.g., by attaching it to a wheelchair), and for safety reasons in case of power cuts.

### 4.2. Respiratory Muscle Training

Respiratory muscle training (RMT) includes strength and endurance training, and has been shown to improve respiratory muscle function in healthy adults, patients with cervical spinal cord injury, and patients with muscular dystrophy [53,54,55]. Several small studies have shown that respiratory muscle strength training (RMST) is feasible and improves respiratory muscle strength in patients with adult Pompe disease and children who survived infantile Pompe disease on ERT [56,57,58,59]. However, evidence is still scarce, and effects of RMT have not yet been investigated using non-volitional measures of respiratory muscle function. As a preliminary recommendation RMST may be carried out on a long-term daily basis using handheld pressure-threshold training devices set to provide a pressure-threshold of 60%–70% of individual MIP and MEP. MIP and MEP should be regularly re-measured and pressure settings adjusted according to treatment success. RMST is not recommended for patients with perforated tympanic membranes, oro-facial weakness, established daytime hypercapnia or thoracic instability. Endurance training as part of RMT has not been investigated in patients with Pompe disease, but preliminary data on other NMD suggest beneficial effects [60,61].

### 4.3. Cough Assistance

As acute respiratory failure (ARF) is a major cause of morbidity and mortality in adult Pompe disease, the management of decreased airway clearance capacity is of utmost importance if expiratory muscle weakness is present. Chest physiotherapy and manually-assisted cough (MAC) may be sufficient only for patients with mild exspiratory muscle weakness. Standard maneuvers include postural drainage and manual techniques (e.g., abdominal thrust maneuvers, thoracic percussion, and special breathing gymnastics). MAC techniques should be implemented by trained physiotherapists or respiratory therapists. MAC efficacy can be improved by voluntary deep breathing or additional techniques which increase expiratory airflow including hyperinflation or air stacking by means of a manual insufflator like a bag valve mask [62] or by glossopharyngeal (“frog”) breathing [63]. In ventilated patients, air stacking can be achieved by applying several mandatory breaths in the volumetric mode while expiration is blocked. Air stacking combined with MAC is generally recommended if cough assistance is indicated and upper airways are patent in cooperative patients [64]. Insufflation/Exsufflation (I/E) devices offer additional cough support by combining full inflation of the lungs and rapid delivery of negative airway pressure, which results in a high peak expiratory flow. I/E devices can be applied either using a face mask or on a tracheostomy tube. They have been shown to improve PCF and airway clearance resulting in significant risk reduction with regard to ARF and hospitalization [65]. High frequency chest wall oscillation (HFCWO) and intrapulmonary percussive ventilation (IPV) promote mucociliary clearance and may help to propel secretions forward from the periphery to the central airways. HFCWO uses an inflatable jacket or cuirass to generate oscillations of the chest wall (5–20 Hz). IPV involves superimposed high frequency mini pressure bursts applied via a nasal-oral mask to create intrapulmonary vibrations. The superiority of either technique has not been demonstrated in NMD [66,67]. Practical recommendations on cough assistance are summarized in Table 3. Contraindications for I/E devices, HFCWO, and IPV include emphysema, chest wall instability, uncontrolled asthma or heart failure, pneumothorax, and pneumomediastinum.

### 4.4. Management of Acute Respiratory Failure

Any condition leading to acute respiratory failure (ARF) in a patient with LOPD is potentially life-threatening. There is a risk of dramatic physical and mental deterioration if ARF is handled without taking Pompe disease into account. Patients are at risk of death or loss of motor abilities that can never be regained. ARF may result from infection, post-surgery complication, or insidious onset progress of respiratory muscle weakness. In addition, acute worsening of hypercapnia may be caused by either the use of respiratory depressants and diuretics, or by oxygen supplementation when NIV has not been established at the same time. Ideally, a multidisciplinary team familiar with both Pompe disease in general and the individual patient in particular will have planned in advance how to avoid ARF and how to manage ARF if it occurs. If possible, elective surgery should be performed at specialized centers in order to avoid secondary transportation. Practical recommendations for management of ARF in adult Pompe disease patients are summarized in Table 4.

### 4.5. Additional Recommendations

#### 4.5.1. Immunizations

Preventing respiratory infections in patients with Pompe disease is clinically important and vaccines play a crucial preventive role. It is recommended that patients with Pompe disease, whether or not they are receiving ERT, receive the same vaccinations as subjects of similar age and comorbidity [69]. Annual influenza vaccination is recommended after emergence of clinically relevant respiratory muscle impairment. Pneumococcal vaccination should be considered mandatory according to standard recommendations.

#### 4.5.2. Obstructive Sleep Apnea

Macroglossia and pharyngeal narrowness are risk factors for obstructive sleep apnea (OSA) [70]. Hypertrophy and weakness of the tongue have been described in adult Pompe disease [71,72] and OSA has been reported in 3 out of 27 patients [28]. Continuous positive airway pressure (CPAP) is the gold standard for treatment, but since it may cause increased diaphragmatic strain, it should be considered only in patients with isolated OSA without signs or symptoms of respiratory muscle weakness. Regular follow-up sleep studies are recommended, and NIV should be started once respiratory muscle weakness is detected.

#### 4.5.3. Concomitant Pulmonary Disease

Concomitant pulmonary disease should be treated according to disease-specific guidelines. Patients with chronic obstructive pulmonary disease (COPD) are at high risk of developing early type 2 respiratory failure since respiratory muscle weakness may be significantly enhanced by chronic hypoxemia, high ventilatory rate, and increased dead space ventilation. Excessive oxygen supplementation may lead to both decreased respiratory drive and worsening of ventilation-perfusion mismatch further promoting hypercapnia. In patients with mild respiratory muscle weakness, REM sleep-associated hypoventilation is much more likely to occur if COPD is present. In summary, in patients with Pompe disease and lung disease we recommend more frequent sleep studies and early initiation of NIV, if indicated. Long-term oxygen treatment should not be implemented without thorough evaluation of respiratory muscle function and nocturnal ventilation, and it is advisable to simultaneously start NIV.

#### 4.5.4. Perioperative Management

Recommendations can only be deduced from single case reports, general considerations on anesthesia in NMD, and own experience. Local anesthesia is generally preferred. If general anesthesia is required, patients should be referred to centers experienced in the perioperative care of patients with NMD. Whereas propofol may be disadvantageous in patients with infantile-onset Pompe disease and cardiomyopathy [73], there is no evidence that it should not be used in patients with LOPD. Both ketamine and etomidate can be safely used for induction of general anesthesia [74]. Patients are more sensitive to neuromuscular blockade, and prolonged weaning should always be anticipated. The perioperative use of opioids may add to this risk by depressing respiratory drive and increasing chest wall rigidity. If scoliosis is present, epidural or spinal anesthesia may be difficult to perform and require special expertise and precautions [75].

#### 4.5.5. Scoliosis

Scoliosis has been reported in one-third of patients with all types of Pompe disease [76] and in 16% of patients with LOPD [77]. Severe scoliosis may impair ventilation increasing the risk of SDB and daytime hypercapnia. Orthopedic management should aim to reduce pain, sitting instability, and lung restriction. Patients should be referred to specialized spine centers. Spinal surgery may be advisable in some patients [78]. Non-surgical treatment includes individualized corsets and long-term physiotherapy.

#### 4.5.6. Nutrition

Patients with adult Pompe disease, particularly with juvenile disease onset, are prone to underweight or even cachexia which may add to respiratory muscle dysfunction [79]. Nutritional counseling should focus on improving BMI, abdominal circumference, body fat content, and hip-waist-ratio. Rarely, percutaneous endoscopic gastrostomy may be considered in patients with advanced disease or severe bulbar dysfunction, respectively.

#### 4.5.7. Chronic Pain

In patients with adult Pompe disease and chronic pain requiring long-term analgesia with opioids, respiratory drive and chest wall compliance may be reduced. Long-term opioid use directly affects sleep architecture and sleep-related breathing [80] making more frequent sleep studies advisable.

#### 4.5.8. Palliative Care

Initiation of palliative care should be considered in patients who have continuous NIV or invasive ventilation without durable improvement or any concomitant fatal disease. There are no specific guidelines for palliative care for patients with Pompe disease or proximal myopathies in general. However, general recommendations can be adopted from guidelines referring to other types of NMD associated with chronic respiratory failure.

#### 4.5.9. Patient and Caregiver Education

Patient and caregiver education with regard to respiratory issues is crucial for recognition and early treatment of any problem related to RMW in patients with Pompe disease. In particular, patients and caregivers should be regularly informed about potential complications, vaccination issues, prevention of ARF, and the importance of sleep-related symptoms as indicators of RMW.

## 5. Methods

The Pompe Respiratory Care Working Group initially met at the 2012 European symposium “Steps Forward in Pompe Disease” (Berlin, Germany) to establish the need for up-to-date expert recommendations on respiratory management of adult patients with Pompe disease. The group began work in 2013 and convened for a two-day meeting in March 2014. Discussions were centered around defining the optimal tests and treatments for respiratory function in terms of clinical validity and relevance, accessibility, cost-effectiveness, and reliability. In addition, for each clinical problem or paraclinical test, any disease-specific evidence available was evaluated. If no evidence with special regard to Pompe disease was obtainable, expert recommendations and clinical guidelines focusing on other NMD, or NMD in general, were consulted. All working group members reviewed and approved the draft and final recommendations.

## 6. Conclusions

Respiratory muscle involvement is a prominent feature of Pompe disease, substantially affecting quality of life, morbidity, and mortality. Thus, adult Pompe disease can be considered a “model disease” for myopathies of adult age, which require close and life-long interdisciplinary co-operation between neurologists and pulmonologists once diagnosis has been established. Although recent pharmacotherapeutic approaches have opened promising perspectives for patients with Pompe disease, respiratory muscle weakness will always affect a large number of patients. Optimal care includes thorough follow-up of respiratory muscle function and sleep-related breathing. Treatment options aim to either delay or compensate for significant respiratory muscle weakness in order to improve overall quality of life and avoid life-threatening complications. Thus, early initiation of adequate treatment is essential, and it is strongly recommended to link patients to specialized centers.

## Figures and Tables

**Table 1:** Practical recommendations for inspiratory and expiratory muscle testing in LOPD. LLN, lower limit of normal; MEP, maximal expiratory pressure; MIP, maximal inspiratory pressure; PCF, peak cough flow; SNIP, sniff nasal inspiratory pressure; TLC, total lung capacity; VC, vital capacity; PFT, pulmonary function testing; IVC, inspiratory vital capacity; SVC, slow vital capacity; ERV, expiratory reserve volume; IRV inspiratory reserve volume; TV, tidal volume; TLC, total lung capacity; RV, residual volume. Normal values are derived from [33].

Test	Device/Method	LLN	Significance	Recommendations
MEP	Manometer	Females 70 cm H_2_O, males 100 cm H_2_O	Expiratory muscle strength	First-line, at least annually
PCF	Peak flow meter	270 L/min, airway clearance impaired if 160–270 L/min, airway clearance impossible if <160 L/min	Reduced vital capacity Reduced inspiratory and expiratory muscle strength	First-line, at least annually widely available
MIP	Manometer	Females 70 cm H_2_O, males 80 cm H_2_O	Inspiratory muscle strength	First-line, at least annually
SNIP	Manometer	Females 60 cm H_2_O, males 70 cm H_2_O	Inspiratory muscle strength	Surrogate of MIP if weakness of the orbicularis oris muscle is present
VC	Spirometry	Upright > 80% of predicted VC, supine > 80% of upright VC	IRV + TV + ERV (global test of lung volume and respiratory muscle performance)	First-line, at least annually

**Table 2:** General indications and contraindications for the initiation of long-term positive pressure ventilation in patients with neuromuscular disease [26]. FVC, forced vital capacity; MIP, maximal inspiratory pressure; SDB, sleep-disordered breathing; paCO_2_, partial pressure of carbon dioxide; RMW, respiratory muscle weakness; saO_2_, oxygen saturation; tcCO_2_, transcutaneous carbon dioxide tension; TIV, tracheostomy invasive ventilation.

Mode	Non-Invasive Ventilation (NIV)	Invasive Ventilation (IV/TIV)
**Indications**	Symptoms of SDB or significant inspiratory muscle weakness	Failure of NIV Persistent NIV intolerance Contraindications to NIV NIV > 20 h/day (consider) Acute respiratory compromise
	and at least one of the following:	
	Daytime hypercapnia (paCO_2_ ≥45 mmHg) Nocturnal hypercapnia (paCO_2_/tcCO_2_ > 50 mmHg) Nocturnal oxygen desaturation (SaO_2_) < 90% for at least five Consecutive minutes Overnight increase of pCO_2_/tcCO_2_ > 10 mmHg from baseline FVC < 50% predicted MIP < 60 cm H_2_O (if rapid deterioration of RMW is present)	
**Contraindications**	Relative	Inadequate caregiver support
	Severe dysphagia Inadequate caregiver support Initial need for full-time ventilation	
	Absolute	
	Persistent upper airway obstruction Persistent hypersecretion Inability to co-operate Inefficient cough (even with assistance)	

**Table 3:** Practical recommendations for cough assistance in patients with LOPD. MAC, manually assisted coughing; I/E, insufflation/exsufflation; HFCWO, high frequency chest wall oscillation; NIV, non-invasive ventilation; TIV, tracheostomy invasive ventilation; PCF, peak cough flow; MEP, maximal expiratory pressure.

**Symptoms**	Mucus obstruction, recurrent desaturations, recurrent pulmonary infections	
**Testing**	PCF, MEP	
**When to start**	PCF < 270 L/min once during stable state independent of symptoms PCF < 160 L/min once during acute exacerbation MEP < 60 cm H_2_O with history of impaired airway clearance	
**Techniques**	MAC	If patient is willing and able to co-operate Performed by respiratory therapists or trained caregivers Re-evaluate feasibility and effectiveness Switch to mechanical techniques if MAC is not feasible or proves ineffective
	Air stacking	Usually in combination with MAC Via bag valve mask in the non-invasive setting Via ventilator device (with NIV or TIV, respectively)
	I/E	If MAC/air stacking are not feasible or ineffective May be combined with MAC Individually titrate optimal pressure settings Feasible in both the NIV and TIV setting Re-evaluate using PCF as outcome measure Start early in case of pulmonary infection
	HFCWO	If MAC/air stacking are either not feasible or ineffective If I/E cannot be tolerated May be combined with MAC Individually titrate frequency and duration Feasible in both the NIV and TIV setting May be combined with suction Start early in case of pulmonary infection
**Optional measures**	Mucolysis	Hydration, mucolytics (with caution)
	Suction	If expectoration cannot be achieved by MAC, I/E, HFCWO alone

**Table 4:** Management of acute respiratory failure (ARF) in patients with LOPD. ERT, enzyme replacement therapy; ICU, intensive care unit; RICU, respiratory intermediate care unit; NIV, non-invasive ventilation; TIV, tracheostomy invasive ventilation; I/E, insufflation/exsufflation; HFCWO, high frequency chest wall oscillation.

Admission to ICU or RICU [68] Broad spectrum antibiotics Avoid opiates and paralytics Always prefer NIV to TIV if possible If TIV is inevitable, aim for early closure of tracheostomy and re-start of NIV Aggressively treat airway secretions (I/E, HFCWO, bronchoscopy) Start respiratory rehabilitation as early as possible Evaluate patients without ventilatory support prior to ARF for NIV indication After rehabilitation, reinforce long-term prophylactic measures (e.g., cough assistance, immunizations) ERT not to be paused

## Abbreviations

ARF	Acute respiratory failure
BE	Base excess
BIPAP	Bilevel inspiratory positive airway pressure
CO_2_	Carbon dioxide
COPD	Chronic obstructive pulmonary disease
CPAP	Continuous positive airway pressure
ERT	Enzyme replacement therapy
ERV	Expiratory reserve volume
FVC	Forced vital capacity
GAA	α-1,4-glucosidase
HFCWO	High frequency chest wall oscillation
IC	Inspiratory capacity
ICU	Intensive care unit
I/E	Insufflation/exsufflation
IPV	Intrapulmonary percussive ventilation
IRV	Inspiratory reserve volume
IVC	Inspiratory vital capacity
LOPD	Late-onset Pompe disease
MAC	Manually assisted coughing
MEP	Maximum expiratory pressure
MIP	Maximum inspiratory pressure
NIV	Non-invasive ventilation
NMD	Neuromuscular disorders
OSA	Obstructive sleep apnea
PCF	Peak cough flow
pCO_2_	Carbon dioxide tension
PFT	Pulmonary function testing
Pdi	Twitch diaphragmatic pressure
Pmo	Twitch mouth pressure
PO	Pulse oximetry
PSG	Polysomnography
REM	Rapid eye movement
RICU	Respiratory intermediate care unit
RMW	Respiratory muscle weakness
RMST	Respiratory muscle strength training
RMT	Respiratory muscle training
RV	Residual volume
SaO_2_	Arterial oxygen saturation
SDB	Sleep-disordered breathing
SNIP	Sniff nasal inspiratory pressure
SpO_2_	Peripheral oxygen saturation
SVC	Slow vital capacity
tcCO_2_	Transcutaneous carbon dioxide tension
TLC	Total lung capacity
TV	Tidal volume
VC	Vital capacity
